# Profiling of Polyphenolic Compounds of *Leontopodium alpinum* Cass Callus Cultures Using UPLC/IM-HRMS and Screening of In Vitro Effects

**DOI:** 10.3390/plants11010100

**Published:** 2021-12-29

**Authors:** Ioana-Ecaterina Pralea, Radu-Cristian Moldovan, Adrian-Bogdan Țigu, Alina-Maria Petrache, Simona-Codruța Hegheș, Monica Mitoi, Gina Cogălniceanu, Cristina-Adela Iuga

**Affiliations:** 1 Research Center for Advanced Medicine—MedFUTURE, Department of Proteomics and Metabolomics, “Iuliu Hațieganu” University of Medicine and Pharmacy Cluj-Napoca, Louis Pasteur Street 6, 400349 Cluj-Napoca, Romania; pralea.ioana@umfcluj.ro (I.-E.P.); moldovan.radu@umfcluj.ro (R.-C.M.); alina.mornea@gmail.com (A.-M.P.); 2 Research Center for Advanced Medicine—MedFUTURE, Department of Translational Medicine, “Iuliu Hațieganu” University of Medicine and Pharmacy Cluj-Napoca, Louis Pasteur Street 6, 400349 Cluj-Napoca, Romania; bogdan.tigu@umfcluj.ro; 3 Department of Pharmaceutical Analysis, Faculty of Pharmacy, Iuliu Hațieganu University of Medicine and Pharmacy, Louis Pasteur Street 6, 400349 Cluj-Napoca, Romania; cmaier@umfcluj.ro; 4 Department of Plant and Animal Cytobiology, Institute of Biology Bucharest, 296 Splaiul Independenței, 060031 Bucharest, Romania; monica.carasan@ibiol.ro (M.M.); gina.cogalniceanu@ibiol.ro (G.C.)

**Keywords:** callus cultures, edelweiss, phytochemical characterization, antioxidants, ion-mobility mass spectrometry

## Abstract

*Leontopodium alpinum* Cass. (edelweiss) is recognized as a frequent constituent of anti-aging skin care products, providing increased antioxidant and anti-inflammatory defense. Considering the growing demand and the protected status of edelweiss in many countries, alternative methods of production have been developed, one of them being callus culturing. This study reports the phytochemical composition of a methanolic extract of *L. alpinum* callus cultures, characterized by liquid chromatography coupled to ion-mobility high resolution mass spectrometry (UPLC/IM-HRMS). The methanolic extract exhibited strong free radical scavenging activity (122.19 ± 7.28 mg AAE/g dw), while the quantitative evaluation revealed that four major constituents (phenylpropanoid derivatives) represent 57.13% (m/m) of the extract. Consequently, a screening of antiproliferative effects was performed on ten cancer cell lines, representative of prostate, colon, lung and breast cancer, showing inhibition of colony formation in all cases. These results provide a comprehensive phytochemical characterization of *L. alpinum* callus cultures using advanced IM-HRMS, while the in vitro explorations confirmed the potent antioxidant properties of edelweiss which are worth exploring further in cancer prevention.

## 1. Introduction

*Leontopodium alpinum* Cass. (edelweiss) is one of the most well-known members of the Asteraceae family. It grows on limestone surfaces, prefers the alpine climate and it is a protected species in many countries. In traditional medicine, edelweiss was used as remedy in digestive and respiratory illnesses as powder, infusion and tincture [1] or in the treatment of breast cancer, applied as a compress [2]. Nowadays, edelweiss is largely used in the cosmetic industry, especially in anti-aging products, for its remarkable antioxidant activity. Considering its protected status and the increased amount required by the industry, alternative technologies have been implemented in order to meet the demand, such as crop cultivation or cell tissue culturing techniques.

Callus culture (of non-differentiated, proliferating and metabolic active cells) is a developing technique for the production of useful compounds, including pharmaceuticals [3]. The most important advantages of in vitro plant cultures are represented by controlled growth environment and nutritive medium, season-independent metabolites production, selection of high-performance cell lines with specific, consistent levels of medicinal compounds, the absence of contaminants, and the possibility of maintaining cells indefinitely by regularly passaging [4]. Small-scale (glass flask) to large-scale (bioreactor) cell cultures have been successfully used for the production of medicinal metabolites [5].

Previous investigations [6,7,8,9,10,11,12] of *Leontopodium alpinum* phytochemical composition highlighted a number of compound classes, such as phenylpropanoid, sesquiterpene, benzofuran and lignan derivatives. However, data regarding the composition of callus cultures is rather limited [13,14]. So far, only the most abundant compounds have been identified, namely derivatives of quinic acid (chlorogenic acid and di-caffeoylquinic acid) and derivatives of glucaric acid (leontopodic acids A and B). The two leontopodic acids are specific to edelweiss and structurally are derivatives of glucaric acid substituted with caffeoyl and 3-hydroxybutanyl moieties [6], showing strong antioxidant potential.

During the last decade, the preclinical research on *L. alpinum* extracts effects has been focused on anti-inflammatory effect evaluation [14], leukotriene biosynthesis inhibition [15], anti-aging effects [16] and memory improving effects [17]. However, no research of edelweiss’s potential in chemoprevention has been published so far. Considering the successful valorization of the antioxidant properties in anti-aging formulations, it is of interest to assess the potential of edelweiss callus cultures in cancer management. Even though until today clinical trials have not demonstrated that dietary antioxidant supplements are beneficial in primary cancer prevention, plants are sources of potential active agents and lead molecules [18], due to the structural diversity of plant secondary metabolites (classes such as flavonoids, terpenes, alkaloids saponins, lignans and other molecules).

The aim of this study was to perform a comprehensive phytochemical characterization of edelweiss callus cultures. Following the assessment of the antioxidant capacity evaluation, two major cancer hallmarks were also investigated (proliferation and resisting cell death) using a panel of ten cancer cell lines representative of breast, prostate, lung and colon cancers.

## 2. Results

### 2.1. Extract Preparation and LC/IM-HRMS Phytochemical Characterization

The *L. alpinum* extract was obtained by microwave assisted extraction (MAE). In comparison to ultrasound assisted extraction (UAE), this technique was found to offer similar yield in a much shorter time (5 min for MAE compared to 45 min by UAE). The total extraction yield was calculated to be 26.4% (i.e., 264 mg of dry extract obtained from 1000 mg of dry frozen callus culture).

**Figure 1:**
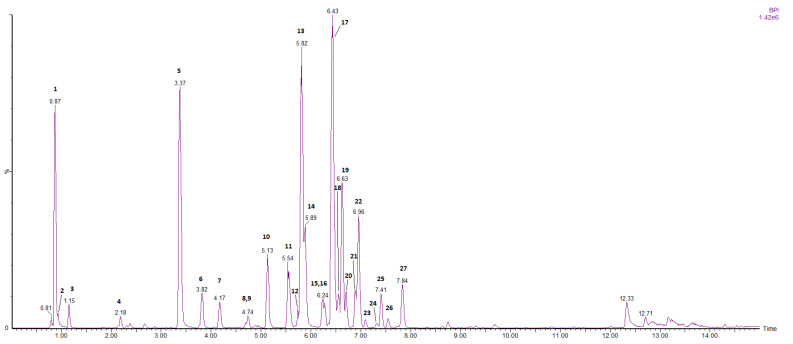
Base peak chromatogram of *L. alpinum* callus cultures’ methanolic extract. Peak numbers correspond to the compounds from Table 1.

Further, the methanolic extract of edelweiss callus cultures was subjected to phytochemical profiling using a reversed phase LC/IM-HRMS method, achieving a good separation of the detected compounds (Figure 1). Considering the structures of the targeted analytes, the detection was performed using negative electrospray ionization followed by ion-mobility mass spectrometry.

Both targeted and untargeted strategies have been implemented to identify most of the compounds present in the extract. The targeted approach was based on literature reports of major phytochemical constituents in *L. alpinum*, whereas for the untargeted approach, a diagnostic product ion (DPI) strategy was implemented [19]. Using this strategy two important classes of derivatives were targeted in *L. alpinum* extract: glucaric acid derivatives and quinic acid derivatives. Therefore, glucaric acid (*m*/*z* 191.0192), peaks **2** and **3** (C_6_H_7_O_7_, 0.3 mDa), and quinic acid (*m*/*z* 191.0556), peak **1** (C_7_H_12_O_6_, 0.6 mDa) were used as DPI (see Supplementary Materials, Figures S1 and S2). Peak **4** was identified as dihydroxybenzoic acid glucoside (C_13_H_16_O_9_, 0.6 mDa) based on the fragmentation pattern and previous reports [20]. Out of a total of 27 detected compounds, 17 were identified with at least level 3 confidence [21] and a mass error below 1.9 mDa. A summary of the identified compounds is presented in Table 1.

#### 2.1.1. Glucaric Acid and Quinic Acid Derivatives

Glucaric acid derivatives are the most abundant class of secondary metabolites identified in *L. alpinum* callus cultures. These derivatives are formed by the esterification of D-glucaric acid hydroxyl groups with caffeoyl and 3-hydroxybutanyl moieties. In total, 11 glucaric derivatives have been identified. Among these, peak **17** leontopodic acid A (C_37_H_34_O_19_, 1.9 mDa) and peak **13** leontopodic acid B (C_33_H_28_O_17_, 0.2 mDa) were the most abundant and their identification was validated using standards. Isomers of both leontopodic A and B were detected as peak **18** and **15**, respectively.

A series of di-caffeoyl glucaric (C_24_H_22_O_14_, 1.2 mDa) isomers were identified as peaks **6, 7** and **8**, while peak **24** represents tetra-caffeoyl glucaric acid (C_42_H_34_O_22_, 0.7 mDa), a tetra-substituted derivative [22]. Considering the fragmentation pattern of peak **26**, it is most likely to be a penta-substituted glucaric acid derivative (C_41_H_40_O_21_, 0.7 mDa), with two 3-hydroxybutanyl and three caffeoyl moieties.

For the identification of quinic acid derivatives, quinic acid (*m*/*z* 191.0556) was used as DPI. Peaks **5** and **14** were identified by comparison with standards and annotated as chlorogenic acid (C_16_H_18_O_9_, 1.3 mDa) and 3,5-dicaffeoyl quinic acid (C_25_H_24_O_12_, 0.2 mDa), respectively. Two other di-caffeoyl quinic isomers (probably 3,4-dicaffeoyl quinic acid and 1,5-dicaffeoylquinic acid) were identified as peaks **12** and **16**.

#### 2.1.2. Quantitative Determinations

LC-MS quantitative determination was made for 4 of the top 5 most abundant identified compounds, i.e., leontopodic acids A and B, chlorogenic acid and 3-5-dicaffeoylquinic acid (Table 2).

The total polyphenol determination revealed that the *L. alpinum* extract contains 230.93 ± 6.6 (mg GAE/g dw), while the in vitro antioxidant activity of the extracts expressed in ascorbic acid equivalent antioxidant capacity (AEAC) was 122.19 ± 7.28 mg AAE/g dw, as determined using the 2,2-Diphenyl-1-picrylhydrazyl (DPPH) free radical scavenging assay.

### 2.2. In Vitro Testing of the L. alpinum Extract

The phytochemical characterization of the LA extract highlighted high concentrations of polyphenolic derivatives with strong antioxidant activities. Consequently, we exposed a panel of diverse cancer cell lines (breast, colon, lung, and prostate cancers) to LA extract and performed several in vitro screenings. Particularly, we focused of two hallmarks of cancer, namely sustaining proliferative signaling (cellular proliferation and colony formation capabilities) and resisting cell death (morphological changes).

#### 2.2.1. Viability Evaluation by MTT Assay

**Figure 2:**
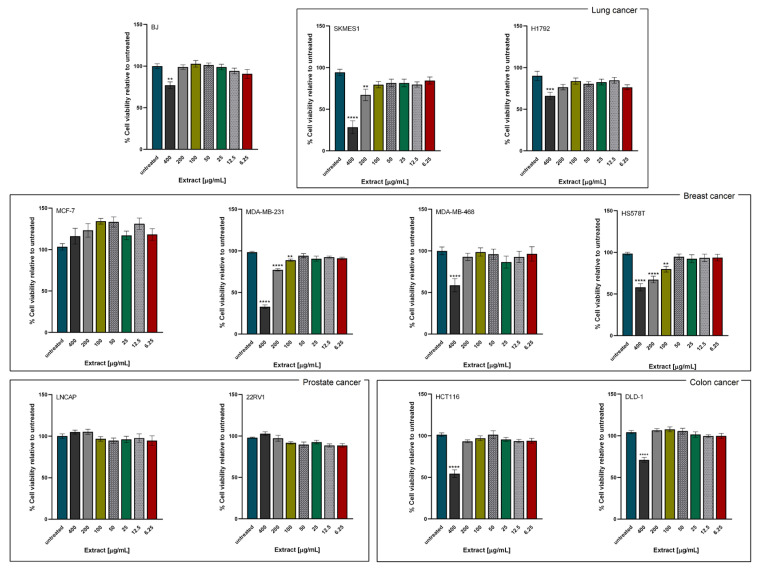
Effects of *L. alpinum* extract on cell viability. The panel of cell lines includes breast, prostate, lung and colon cancer representatives, as well as human fibroblast BJ. Cells were exposed to the indicated concentrations of *L. alpinum* extract for 48 h. Untreated cells (blank control) were exposed only to culture media while positive controls were represented by cells treated with 100% DMSO. Cell viabilities are expressed as percentage of control (set as 100%) and represented as mean ± SEM (*n* = 3). Data was analyzed by one-way ANOVA followed by Dunnett’s multiple-comparison test. The asterisks *, **, *** and **** indicate significant difference at *p* < 0.05, *p* < 0.01, *p* < 0.001 and *p* < 0.0001, respectively, compared to corresponding control.

Effect of *L. alpinum* extract on cancer cell panel viability was studied by 3-(4,5-dimethylthiazol-2-yl)-2,5-diphenyltetrazolium bromide (MTT) assay. The assay involves conversion of 3-(4, 5-dimethylthiazol-2-yl)-2,5-diphenyltetrazolium bromide by NADPH-dependent oxidoreductases to a purple-colored formazan product whose VIS absorbance is proportional to the number of metabolic active cells. Cells were grown in 96-well plates and treated with complete medium containing serial dilutions of extract (6.25–400 µg/mL). A blank control (complete medium only) and a positive control (100% DMSO) were included in every plate. The extract showed significant effect on cell viability at the highest tested concentration (400 µg/mL) on most cell lines (Figure 2), excepting estrogen positive MCF-7 breast cancer line and both androgen receptor positive LNCAP and 22RV1 prostate cancer cell lines. SK-MES-1 lung cancer representative and TNBC MDA-MB-231 cell lines had their viability diminished less than 50% by the LA extract while relative viability of H1792 lung cancer and TNBC HS578T cell lines was 66.2 ± 13.8% and 58.1 ± 13.4%, respectively. Similarly, the percentage of viable colon cancer cells HCT116 and DLD-1 after 48 h of treatment with 400 µg/mL *L. alpinum* extract decreased to 54.5 ± 14.8% and 71.1 ± 9.2%, correspondingly. Furthermore, 400 µg/mL of LA extract reduced viability in the non-cancer human fibroblast cell line BJ to 77.0 ± 9.7%. In addition, some of the cell lines had a significantly impaired viability compared to control at lower doses of LA extract (200 µg/mL): this is the case for SK-MES-1 (67.2 ± 23.8%) lung cancer cell line and MDA-MB-231 (77.21 ± 4.9%) and HS578T (67.4 ± 13.3%) TNBC representatives.

The 400 µg/mL dose of *L. alpinum* extract had the greatest impact on cell viability and was used in the in vitro functional assays conducted further.

#### 2.2.2. Nucleus and the Cytoskeleton Morphological Analysis

The effect of the *L. alpinum* callus cultures’ methanolic extract on cellular morphology and cell distribution was evaluated by 4′,6-diamidino-2-phenylindole (DAPI) and Fluorescein phalloidin (Phalloidin-FITC) staining, used for nuclei and actin filaments labeling. The levels of toxicity and the antiproliferative effects were assessed by comparing control and treated cells groups. A summary of observed effects is found in Table 3, while the microscopy images are available in Supplementary Materials, Figure S3.

A substantial reduction of cell population was seen in the human non-cancer fibroblast (BJ cell line) and MDA-MB-231 TNBC in accordance with the viability assay conducted. Reduction of cell population was also observed in the case of both HCT-116 and DLD-1 colon cell lines. Particular to BJ cell line was the absence of signs of nuclear and cytoskeletal damage.

The *L. alpinum* extract induced nuclear fragmentation on most cell lines, excepting BJ, HS578T and SK-MES-1. Furthermore, multilobed nuclei were observed for HS578T, SK-MES-1, 22RV1, and HCT116, associated with rounding of cells. This last phenomenon was found also for H1792 and LNCAP in a less extensive manner. A marked cytoskeleton fragmentation was observed in the case of prostate cancer compared to the other cell lines.

Compared to TNBC cell lines, MCF-7 was less sensitive to *L. alpinum* extract, few cells presenting nuclear fragmentation and cytoskeleton damage, with most of the cells having similar shape to the control.

#### 2.2.3. Clonogenic Cell Survival Assay

Another cancer feature is the capacity to develop secondary cancer sites, also known as metastatic sites. Colony assay was chosen to analyze this ability in vitro, by measuring the colony diameter of both control and treated cells (Figure 2 and microscopy captures in Supplementary Materials, Figure S4).

**Figure 3:**
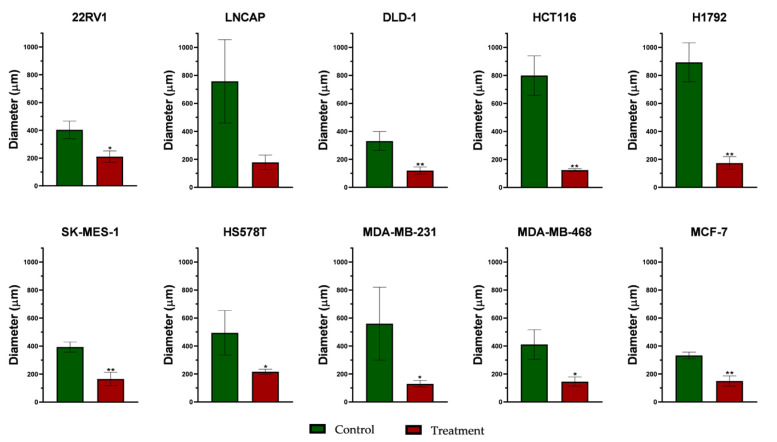
Colony assay evaluation on ten cancer cell lines. (Statistical analysis: unpaired *t*-test was applied to determine significant differences in colony numbers. * *p* < 0.05, ** *p* < 0.01).

After treatment, 22RV1 and LNCAP prostate cells colonies were considerably reduced and both cell lines exhibited a low ability to create new colonies (Figure 3). Compared to the other cancer cell lines, exposing these cells to *L. alpinum* extract generated small and hardly visible colonies in the captures, the colony formation capacity of 22RV1 being half of control, while for LNCAP cells the capacity was even lower.

HCT116 colon cancer cells showed higher capacity to form colonies compared to DLD-1. The extract considerably inhibited the colony formation of both cell lines, with a pronounced effect on HCT116. Lung cancer cell lines (H1792 and SK-MES-1) showed high colony formation capacity, but the treatment reduced the colony number and size. The inhibitory effect of the treatment was confirmed on all three cell lines, the colony diameter being decreased by more than half compared to that of control. Similar to lung, colon and prostate cell lines, the extract inhibited colony formation was seen in all breast cancer cell lines. Breast cancer cell lines exhibited different colony formation capacities, the most potent being MCF-7, followed by MDA-MB-468 and the other two TNBC cell lines.

## 3. Discussion

In traditional medicine, *L. alpinum* was used mostly for its anti-inflammatory properties. Today, its current use is limited due to the protected species status that the plant has in many countries. However, the growing demand coming from the cosmetic industry, due to the antioxidant properties of *L. alpinum,* required the development of alternative means to obtain the specific constituents, such as callus cultures of edelweiss, now a world-wide substitute.

In medicine, many plant antioxidants have been shown to possess antitumoral properties [23]. Although edelweiss has been known to possess strong antioxidant properties, to our best knowledge no study has aimed to investigate/harness these properties in cancer research.

Microwave assisted extraction (MAE) and ultrasound assisted extraction (UAE) were assessed for the targeted extraction of polyphenolic compounds from edelweiss callus cultures, using the same extraction solvent (70% methanol). MAE was found to offer a faster extraction time (5 min), compared to UAE (45 min), both having comparable yield (data not shown). The dried extract was easily soluble in aqueous solutions, even at high concentrations (4 mg/mL stock solutions).

The polyphenol profiling of the *L. alpinum* extract was performed using LC/IM-HRMS. Compared to conventional mass spectrometry, ion-mobility mass spectrometry offers a supplementary separation dimension. Being a gas phase separation technique, ions are resolved based on their size and shape, depending on their collisional cross section (CCS). Ion-mobility mass spectrometry proved to be useful for differentiating between isomeric species [24]. Still, for the analysis of *L. alpinum* extract, no co-eluting isomeric species could be identified, all of them being resolved chromatographically before detection. The CCS values of the molecular ions of the constituents detected in the extract are presented in Table 1. As expected, small CCS differences were observed for the di-caffeoyl-glucaric acid isomers (peaks **6**, **7** and **8**) and di-caffeoyl quinic acid isomers (peaks **12**, **14**, **16**). However, their separation by ion-mobility alone would probably not be possible considering the low resolution of today’s commercially available technology.

The analysis of the extract revealed 27 major constituents. The two detected classes of polyphenols were derivatives of glucaric and quinic acids, the most abundant being leontopodic acids A and B, chlorogenic acid and 3,5-dicaffeoyl quinic acid. Besides these already known constituents, several other derivatives were observed (some of which are reported for the first time in *L. alpinum*) such as isomers of leontopodic acids A and B, disubstituted derivatives of both glucaric (di-caffeoyl glucaric acids) and quinic acids (di-caffeoyl quinic acids), together with two highly substituted derivatives of glucaric acids (tetra-caffeoyl glucaric acid and a penta-substituted glucaric acid derivative).

Even though most of the detected compounds could be identified with a high degree of certitude, ten compounds remain unidentified. Most of these do not possess any similarity (molecular ion or fragment ions) with other compounds reported in the composition of *L. alpinum*, such as terpenes [9,10,11], lignans [25], or flavonoids [7].

Quantitative analysis of the most abundant four phenylpropanoid derivatives specific to *L. alpinum* (i.e., chlorogenic acid, 3,5-dicaffeoyl quinic acid, leontopodic acids A and B) (Table 2) revealed that they represent more than 57% (m/m) of the dry-frozen extract and around 15% (m/m) of the dry-frozen callus culture. The measured concentrations of these four constituents in callus culture extracts are comparable or higher than in wild plant [16] and confirm the high quantity of polyphenols obtained by the total polyphenol content determination. Therefore, they are expected to be responsible for the effects observed in the in vitro assays that were carried out. The extract also showed strong antioxidant activity as determined through the DPPH free radical scavenging assay, in agreement with previous studies [13,16].

Considering the high quantity of polyphenols determined in the edelweiss callus cultures, an in vitro screening procedure was implemented which allowed the evaluation of the extract on three cancer hallmarks. Its effects were studied on ten cancer cell lines, representative of four different cancer types. A summary of the obtained results is presented in Table 3.

The cytotoxic effect of *L. alpinum* callus cultures extract was evaluated in vitro using the MTT assay. Decreased cell viability was observed at the highest concentration tested (400 µg/mL) for TNBC breast cell lines MDA-MB-231 and HS578T, both colon (DLD-1, HCT-116), and in lung cancer cell lines (SK-MES-1 and H1792). In the specific case of MCF-7, an estrogen dependent cell line, pro-proliferative effects have been observed. It has already been documented that polyphenols, due to their chemical structure, can interact with estrogen receptors with different affinity towards estrogen receptors α and β. A deeper insight into the mechanisms involving polyphenols’ effects on hormone dependent cancer cell lines can be found in a comprehensive review published by Cipolletti et al. [26].

Next, other cancer hallmarks were studied in order to observe potential induced morphological changes and colony formation capabilities. Colony formation was inhibited for all cell lines, presenting substantially reduced diameters (Figure 2), with pronounced inhibition being observed on HCT116, H1792 and MDA-MB-231 cell lines, both assays being in accordance with the morphological analysis (see Supplementary Materials, Figure S3). The extract induced morphological alterations, major differences being observed between control and treated groups on several cell lines: an augmented nuclear fragmentation was observed in 22RV1 and HCT116, while the prostate cancer cells presented a distorted cytoskeleton. Rounding of cells was observed in HS578T, SK-MES-1 and less extensively in H1792 and HCT-116. On the other hand, MDA-MB-468 and MCF-7 cell lines were less affected by the treatment, cell populations not being considerably reduced, with fewer nuclear fragmentations. Membrane blebbing was be observed on MDA-MB-468 as a sign of cellular stress, but without effect on cell population.

These results could be attributed to the high quantity of polyphenols which are known to modulate pathways relevant to proliferation, migration or colony formation and in some particular cases cell death could be triggered [27,28,29,30].

Using in vitro models, polyphenols were previously described to possess anticarcinogenic properties, capable of inhibiting tumor growth, metastasis and inflammatory processes [31,32,33]. The major constituents of the edelweiss callus culture extract have been previously reported to possess strong antioxidant properties, protecting cells against DNA damage [6]. For example, chlorogenic acid, one of the major constituents of the extract, can affect GSK-3β, APC and β-catenin gene expression acting as an antitumor agent [34]. It was also described that it is able to interact with key molecules that can further modulate critical biological pathways involved in cell survival or proliferation [34]. It was also shown that chlorogenic acid can enhance the antitumor effect of conventional therapeutic agents like doxorubicin by protecting the non-cancer cells against doxorubicin toxicity [35] or induce hypomethylation of Jurkat cells, being benefic in the case of hematological malignancies [27].

The second quinic acid derivative found in high quantities in *L. alpinum* extract, 3-5-dicaffeoylquinic acid, acts as antioxidant agent with neuroprotective effects [36]. On top, 3,5-dicaffeoylquinic acid was shown to protect cardiomyocytes cell line from apoptosis by increasing activation of the PI3K/Akt signaling pathway [37]. The antiproliferative effect of several di-caffeoylquinic acids including 3,5-dicaffeoylquinic acid was demonstrated on MCF-7 and MDA-MB-231 breast cancer cells with reported IC_50_ below 300 µM (MTT assay, 48 h treatment) [38]. The authors reported 1,3-dicaffeoylquinic acid as potent inhibitor of 14-3-3τ protein, thus preventing breast cancer proliferation and metastasis via Jak/PI3K/Akt and Raf/ERK pathway.

Unfortunately, less is known about the potential anticancer mechanisms of actions of the leontopodic acids A and B, the other two major constituents of *L.alpinum* callus cultures, and remains a subject open to research.

## 4. Materials and Methods

### 4.1. Chemicals and Reagents 

The reference standards of chlorogenic acid, 3,5-dicaffeoylquinic acid and leontopodic acids A and B which were used in this work were acquired from Extrasynthese (Genay, France). The organic solvents, methanol and acetonitrile, used for mobile phases and extractions were of LC-MS purity and were purchased from Merck (Darmstadt, Germany). Ultrapure water was produced with a Purelab water filtration system (Elga LabWater, Celle, Germany), while MS grade formic acid was produced by Carlo Erba (Val de Reuil, France). 2,2-Diphenyl-1-picrylhydrazyl and Folin-Ciocâlteu’s phenol reagent were purchased from Sigma (Steinheim, Germany), ascorbic acid was produced by International Laboratory (Cluj-Napoca, Romania), and sodium carbonate by Lachner (Neratovice, Czechia).

### 4.2. Biological Material

Commercial seeds of *Leontopodium alpinum* Cass. (www.semintelegumeflori.ro (accessed on 10 December 2021), batch Pl730/12/210/1274A, SC XTREME ANG MARKETING SRL, Bucharest, Romania), aseptically germinated, and seedlings were used to initiate the callus culture on Gamborg medium [39], supplemented with 2,4-dichlorophenoxyacetic acid, 6-benzylaminopurine, indole-3-acetic acid, sucrose, casein hydrolisate, calcium carbonate and agar. The cultures were grown in a Fitotron Weiss-Gallenkamp SCG 120 (Weiss Technik, Loughborough, UK) with a photoperiod of 16 h light/8 h dark, at a temperature of 25 °C and sub-cultivated on the same fresh medium every 3 weeks. (Patent pending).

### 4.3. Extract Preparation

Prior to extraction, *L. alpinum* callus cultures from three batches were freeze-dried (0.5 mbar, −55 °C) using a LyoQuest -55 Plus instrument (Telstar, Terrassa, Spain), then all samples were mixed to create a representative sample which was further used for this study. Ultrasound assisted extraction (UAE) and microwave assisted extraction (MAE) were assessed in order to find the most suitable extraction conditions.

For UAE, 20 mg of freeze-dried sample was suspended in 1 mL of 70% methanol (*v*/*v*), then sonicated for 45 min at 37 kHz (Elmasonic S 30H, Elma, Singen, Germany) [19]. MAE (by means of Multiwave PRO (Anton Paar, Graz, Austria)) was performed starting from 120 mg of freeze-dried sample which was placed into the extraction vessel and suspended in 6 mL of 70% methanol (*v*/*v*) [40]. The experiment was carried at 60 °C for 5 min, the microwave power varying up to 500 W [41,42,43]. Then, the extracts obtained by both UAE and MAE were centrifuged for 10 min at 8000 rpm and liquid-liquid extraction with n-hexane 1:1 (*v*/*v*) was performed 3 times (for removal of lipid components), the organic phase being discarded [44]. The methanol phase was evaporated using a rotary evaporator (40 °C, 150 mbar) (Heidolph Instruments, Schwabach, Germany), then the remaining aqueous phase was freeze-dried (0.5 mbar, −55 °C).

### 4.4. Chromatographic Method and Instrumentation

All analyses performed for the phytochemical characterization were carried out on a Waters I-Class UPLC instrument coupled with a Synapt G2-Si high resolution mass spectrometer (Waters, Milford, MA, USA). The separations were achieved by reversed phase using a BEH C18 (100 × 2.1 mm) stationary phase (Waters, Milford, MA, USA) and mobile phases formed of 0.1% formic acid in water (A) and 0.1% formic acid in acetonitrile (B), being pumped at a flow rate of 0.3 mL/min at a temperature of 45 °C. Gradient elution was implemented as follows: 0 min 3%B–10 min 40%B–15 min 98%B. The mass spectrometer was set in resolution mode using negative electrospray ionization (2 kV capillary voltage), with source temperature of 130 °C. Data independent acquisition was achieved in two modes: (i) HDMSE (ion-mobility enabled) for qualitative runs and identification purposes and (ii) MSE (Q-ToF only) for quantification. Data was acquired in the range of 50 to 1200 *m*/*z*, using 2 functions. The collision energies were set to 4 eV (trap) and 2 eV (transfer) for the first function and a ramp between 20–45 eV (trap) for the second function. Leucine-encephalin was used as single point reference (*m*/*z* 554.2615).

Data processing (peak picking, alignment) was carried out using Progenesis QI software version 3.0.76 (Waters, Milford, MA, USA). A first identification was performed on the most abundant compounds by using an in-house natural compounds library. Major compounds were putatively annotated based on their accurate mass, fragmentation pattern, available reference standards and on other previous studies.

Quantitative determinations of leontopodic acids A and B, chlorogenic acid and 3-5-dicaffeoylquinic acid were made by constructing a 5-point calibration curve (0.1–40 µg/mL) for each analyte. Each standard solution was injected 2 times and the quantitative analysis was performed using the TargetLynx module of MassLynx software (Waters, Milford, MA, USA), by measuring the molecular peak areas of the analytes. Injection repeatability was assessed by injecting 5 times the previously mentioned standards (RSD less than 3.85%). Two replicates of the extract were prepared, then analyzed twice, after a 40-fold dilution.

### 4.5. Antioxidant Activity through DPPH Free Radical Scavenging Assay

Antioxidant assay was performed using a microplate protocol described by Navarro et al. [45]. Two series of dilutions of extract (1.56–500 µg/mL) and ascorbic acid (0.19–50 µg/mL) were prepared. 20 μL of sample was added to 180 μL of DPPH solution (150 μM) in methanol–water (80:20, *v*/*v*) and shaken for 60 s. The mixtures were kept for 40 min in darkness at room temperature, then absorbance at λ = 515 nm was measured using a Clariostar Plus microplate reader (BMG Labtech, Ortenberg, Germany). The blank sample consisted of 20 μL of water with 180 μL of methanol:water (80:20, *v*/*v*), while the control sample was 20 μL of water with 180 μL of DPPH solution. Each determination was performed in triplicate and the percentage of quenched DPPH was determined using Equation (1).

(1) $$\%DPPH\;quenched=\left[1-\left(\frac{A_{sample}-A_{blank}}{A_{control}-A_{blank}}\right)\right]\;\times \;100$$

By calculating the concentrations of sample and extracts equivalent to 50% quenched DPPH, the ascorbic acid equivalent antioxidant capacity was determined.

### 4.6. Total Polyphenols Content Determination

The total polyphenols determination was made according to the method described by Singleton et al. [46] and modified by Mihailovic et al. [47], adapted to microplate determinations. In brief, 100 µL of Folin-Ciocâlteu reagent (diluted 4-fold) was added to 20 µL of extract solution or gallic acid standards (10–200 µg/mL). After 3 min, 75 µL of Na_2_CO_3_ was added, followed by incubation at room temperature and darkness for 2 h. The solution absorbance was measured at λ = 765 nm (Clariostar Plus, BMG Labtech, Ortenberg, Germany). The determination was made in triplicate. The gallic acid calibration curve was built, showing a linear range for concentrations between 10–200 μg/mL gallic acid. The total polyphenol content of extracts was expressed as mg of gallic acid equivalents (GAE)/g dry weight (dw), using the Equation (2) based on the calibration curve [48].

(2) $$T=\frac{C\;\times \;V}{M}$$

where, *T* = the total phenolic content in mg/mL as GAE; *C* = the concentration of gallic acid from the calibration curve in mg/mL; *V* = the volume of the extract solution in mL; *M* = the weight of the extract in g.

### 4.7. Cell Cultures and Assays

A panel consisting of twelve human cell lines was chosen for the in vitro evaluation of *L. alpinum* extract. Non-cancer human epithelial fibroblast (BJ—ATCC CRL-2522) and double positive breast cancer cell line (MCF7—ATCC HTB-22) were cultivated in MEM supplemented with 10% Fetal Bovine Serum (FBS) and 1% Penicillin/Streptomycin. Triple negative breast cancer cell lines (MDA-MB-231—ATCC HBT-26, MDA-MB-468—ATCC HBT-132 and HS578T—ATCC HBT-126), lung cancer cell lines (H1792—ATCC CRL-5895 and SK-MES-1—ATCC HTB-58), colon cancer cell line DLD-1 (ATCC CCL-221) and prostate cancer cell lines (LNCAP—ATCC CRL-1740 and 22RV1 ATCC CRL-2505) were maintained in RPMI supplemented with 10% FBS, 1% Penicillin/Streptomycin and 1% Glutamine, while HCT116 colon cancer cell line (ATCC CCL-247) was cultivated in McCoy’s supplemented with 10% FBS and 1% Penicillin/Streptomycin. All cell lines were maintained in a humidified chamber, at 37 °C and 5% CO_2_. All the reagents needed for cell culturing were purchased from Gibco (Dublin, Ireland).

The *L. alpinum* extract stock solution was prepared at a concentration of 4 mg/mL by dissolving the necessary amount of freeze-dried extract in the appropriate culture medium for each cell line. Subsequent dilutions were prepared at specific concentrations needed for the different assays.

#### 4.7.1. Cell Viability Testing by MTT Assay

The effect of *L. alpinum* callus cultures’ methanolic extract was investigated using the MTT assay. Cells were grown at a density of 1 × 10^4^ cells/well in a 96-well flat-bottom plate, each treatment having 3 replicates. After 24 h incubation, cells were treated with different concentrations of extract (6.25–400 μg/mL) for 48 h. To remove possible extract residues that might interfere with MTT assay, a wash step with Phosphate Buffer Saline 1× (PBS) was performed, then cell viability was determined after incubation for 3 h with 100 μL 1 mg/mL MTT. Afterwards, the solution was removed and 150 μL of DMSO were added to dissolve the crystals. The absorbance corresponding to the viable cells was measured at λ = 570 nm on a SPARK10M multiplate reader (Tecan, Männedorf, Switzerland). A blank control (medium only), and a positive control (DMSO 100%), were included on every plate. The experiment was repeated three times. Statistical analysis was performed using Graphpad Prism (version 8) software (GraphPad Software, San Diego, CA, USA). Cell viabilities were expressed as percentage of control (set as 100%) and represented as mean ± SEM. Data was analyzed by one-way ANOVA followed by Dunnett’s multiple-comparison test.

#### 4.7.2. Colony Forming Assay

An equal number of cells (500 cells/well) were seeded in a 6-well plate, both for control and treatment. After 24 h the cells were treated with *L. alpinum* extract (400 µg/mL) and incubated for 48 h, then the cell culture medium was replaced with treatment free medium. Cells were observed daily up to 14 days and when the colonies reached the proper size (more than 50 cells/colony) or when the colonies were too close to another, the experiment was stopped and the cells were fixed with methanol and stained with crystal violet (Sigma-Aldrich, St. Louis, MO, USA) [49,50]. The colonies were observed using a Zeiss Axio Vert A1 inverted microscope (Zeiss, Jena, Germany), colonies diameter being determined using ZEN software (Zeiss, Jena, Germany), by measuring the diameter of three random colonies. Statistical analysis was done on Graphpad Prism (version 8) software (GraphPad Software, San Diego, CA, USA). An unpaired *t*-test was applied to determine significant differences in colony numbers. *

#### 4.7.3. Morphological Analysis

To investigate the morphological changes induced by the 48 h treatment with *L. alpinum* extract (400 µg/mL), cells were analyzed in bright field with an inverted microscope. The same instrument was used in combination with a fluorescent source (HBO50, same manufacturer) for details regarding the cytoskeleton and nucleus, after staining these cell compartments with Phalloidin-FITC (for the cytoskeleton) (Cytoskeleton, Denver, CO, USA) and DAPI (for the nucleus) (Thermo Fisher Scientific, Waltham, MS, USA) using a previously published protocol [50].

## 5. Conclusions

The phytochemical profiling of *L. alpinum* callus cultures revealed high quantities of polyphenols, most of them being derivatives of glucaric and quinic acids. The four most abundant represent more than 57% (m/m) of the dry-frozen extract and around 15% (m/m) of the dry-frozen callus culture, demonstrating that in vitro culturing can be a useful alternative to produce natural compounds.

The screening of cancer cell lines revealed that the *L. alpinum* callus cultures extract demonstrated interesting effects regarding its capacity to inhibit proliferation and colony formation. *L. alpinum* callus cultures extract showed considerable inhibitory effect on SK-MES-1, MDA-MB-231 and HCT116 cell lines, most probably being related to the high content in polyphenolic compounds which exhibit strong antioxidant capacity.

Considering its abilities to inhibit cancer proliferation and colony formation, the use of *L. alpinum* as chemosensitizer might represent a perspective. To better understand the possible effects of leontopodic acids A and B, further research could also focus on evaluating the proliferation, migration or colony formation assays on cancer cells treated with these two natural products.

## 6. Patents

Cogălniceanu G. C., Mitoi E. M., Ciocan A. G., Holobiuc M. I., Maximilian R. C., Helepciuc F. E., Morosanu A. M. Biotechnological procedure for initiating and obtaining high proliferative cell mass producing bioactive compounds in *Leontopodium alpinum* Cass. (Edelweiss) and the crude extract, OSIM Romania Patent Application A/00740/17.11.2020.

## Figures and Tables

**Table 1:** Compound identifications obtained after LC-IM-MS analysis of *L. alpinum* callus cultures.

Peak	Rt (Min)	*m/z* Measured (Da)	*m/z* Calculated (Da)	ΔmDa	Formula [M − H]^−^	Major Fragments	CCS (Å^2^)	Tentative Identification	Identif. Level *
**1**	0.87	191.0561	191.0556	0.6	C_7_H_11_O_6_	-	113.55	Quinic acid	2
**2**	0.93	191.0195	191.0192	0.3	C_6_H_7_O_7_	111.0085	113.23	Glucaric acid—(H_2_O)	2
**3**	1.15	191.0195	191.0192	0.3	C_6_H_7_O_7_	111.0086	113.25	Glucaric acid isomer—(H_2_O)	2
**4**	2.18	315.0722	315.0716	0.6	C_13_H_15_O_9_	152.0109, 108.0214	161.99	Dihydroxybenzoic acid glucoside	2
**5**	3.37	353.0886	353.0873	1.3	C_16_H_17_O_9_	191.0561	171.81	Chlorogenic acid **	1
**6**	3.82	533.0943	533.0931	1.2	C_24_H_21_O_14_	371.0618, 191.0195	206.43	Di-caffeoyl-glucaric acid isomer	2
**7**	4.17	533.0943	533.0931	1.2	C_24_H_21_O_14_	371.0617, 191.0195	207.06	Di-caffeoyl-glucaric acid isomer	2
**8**	4.70	533.0941	533.0931	1.0	C_24_H_21_O_14_	371.0541, 191.0195	209.91	Di-caffeoyl-glucaric acid isomer	2
**9**	4.74	441.1396	-	-	-	431.1111, 395.1347, 233.0822	196.85	Unidentified	-
**10**	5.13	441.1396	-	-	-	431.1111, 395.1353, 233.0823	194.03	Unidentified	-
**11**	5.54	447.1333	-	-	-	267.0688, 187.1081, 132.0561	190.84	Unidentified	-
**12**	5.74	515.1194	515.1190	0.4	C_25_H_23_O_12_	353.0869, 191.0540	207.25	Di-caffeoyl quinic acid isomer	2
**13**	5.82	695.1250	695.1248	0.2	C_33_H_27_O_17_	533.094, 371.0616, 209.0295	235.82	Leontopodic acid B **	1
**14**	5.89	515.1192	515.1190	0.2	C_25_H_23_O_12_	353.0884, 191.0559	206.29	3-5-Dicaffeoylquinic acid **	1
**15**	6.24	695.1256	695.1248	0.8	C_33_H_27_O_17_	533.0953, 371.0609, 209.0291	237.11	Leontopodic acid B isomer	2
**16**	6.27	515.1191	515.1190	0.1	C_25_H_23_O_12_	353.0869	210.01	Di-caffeoylquinic acid isomer	2
**17**	6.43	781.1635	781.1616	1.9	C_37_H_33_O_19_	619.1305, 457.0991, 295.0668, 191.0195, 161.0241	252.37	Leontopodic acid A **	1
**18**	6.55	781.1635	781.1616	1.9	C_37_H_33_O_19_	619.1305, 457.0991, 295.0668, 191.0195, 161.0241	254.65	Leontopodic acid A isomer	2
**19**	6.63	687.2325	-	-	-	643.2434, 625.2344, 525.1796	245.99	Unidentified	-
**20**	6.70	285.0806	-	-	-	187.1138, 132.0585	159.91	Unidentified	-
**21**	6.94	689.2512	-	-	-	645.2613, 627.2507	246.59	Unidentified	-
**22**	6.96	689.2524	-	-	-	645.2619, 627.2514	245.98	Unidentified	-
**23**	7.09	311.0596	-	-	-	231.1030, 187.1129, 132.0581	167.45	Unidentified	-
**24**	7.33	857.1572	857.1565	0.7	C_42_H_33_O_22_	695.1257, 533.0953, 371.0629, 209.0308, 191.0201	261.88	Tetra-caffeoyl glucaric acid	2
**25**	7.41	607.2763	-	-		563.2867, 463.2362, 301.1818, 209.0307	242.57	Unidentified	-
**26**	7.55	867.1991	867.1984	0.7	C_41_H_39_O_21_	705.1687, 543.1373, 381.1049, 277.0576, 209.0280, 191.0202	270.18	Penta-substituted derivative of glucaric acid	3
**27**	7.84	283.0646	-	-	-	203.1081, 148.0531	156.10	Unidentified	-

* Identification level confidence (as described by Schrimpe-Rutledge et al. [21]); ** Identified by comparison with reference standards.

**Table 2:** Quantitative determination of the major constituents.

Compound	µg/mg of Dry Extract	mg/g of Dry-Frozen Callus Culture
**Leontopodic acid A**	178.6	47.15
**Leontopodic acid B**	219.9	58.05
**Chlorogenic acid**	99.1	26.16
**3-5-Dicaffeoylquinic acid**	73.7	19.45
**Total**	**571.3**	**150.81**

**Table 3:** Summary of the in vitro effects of *Leontopodium alpinum* treatment on the investigated cell lines.

Cancer Type			Prostate		Colon		Lung		Breast			
**Cell line**		BJ	22RV1	LNCAP	DLD-1	HCT-116	H1792	SK-MES-1	MDA-MB-231	MDA-MB-468	HS578T	MCF-7
**Viability assay**		+	n.c.	n.c.	++	++	+	+++	+++	++	++	n.c.
**Morphology**	Cell population reduction	++	+	-	+++	+++	++	+	+++	-	+	-
	Nuclear fragmentation	-	+++	+	++	++	+	-	++	+	+	++
	Cytoskeleton fragmentation	-	++	++	++	-	+++	+	+	+	++	++
	Rounding of cells	-	++	++	++	+++	+++	+++	-	-	++	+
	Multi-lobed nuclei	-	+	-	-	+	-	+	-	-	+	-
**Colony assay**		n.d.	+	+	+	+	+	+	+	+	+	+

n.c.—no correlation; “+”—small effect; “++”—medium effect; “+++”—considerable effect; “-”—effect absent; n.d.—no data.

## Data Availability

All data used in and created by this study are included in this publication as tables, figures, and Supplementary Files.

## Supplementary Materials

The following are available online at https://www.mdpi.com/article/10.3390/plants11010100/s1, Figure S1. Diagnostic product ion—glucaric acid; Figure S2. Diagnostic product ion—quinic acid; Figure S3. Morphological analysis; Figure S4. Colony assay.
