# Supplementary material for: Profiling of Polyphenolic Compounds of Leontopodium alpinum Cass Callus Cultures Using UPLC/IM-HRMS and Screening of In Vitro Effects
Source: Plants (Basel). 2021 Dec 29;11(1):100. doi: 10.3390/plants11010100 (PMC8747157; doi:10.3390/plants11010100)
Supplement: Supplementary file 1 [file plants-11-00100-s001.zip › plants-1531420-supplementary.pdf]

Supplementary Information for:

Profiling of polyphenolic compounds of *Leontopodium alpinum* Cass. callus cultures using UPLC/ESI-IM-HRMS and screening of *in-vitro* effects

Ioana-Ecaterina Pralea[1,†] , Radu-Cristian Moldovan[1,†], Adrian Bogdan Țigu[2], Alina-Maria Petrache[1], Simona-Codruța Hegheș[3], Monica Mitoi[4], Gina Cogălniceanu[4], Cristina-Adela Iuga[1,3,*]

[1] Department of Proteomics and Metabolomics, Research Center for Advanced Medicine – MedFUTURE, ”Iuliu Hațieganu” University of Medicine and Pharmacy Cluj-Napoca, Louis Pasteur Street 6, 400349 Cluj-Napoca, Romania;

[2] Department of Translational Medicine, Research Center for Advanced Medicine – MedFUTURE, ”Iuliu Hațieganu” University of Medicine and Pharmacy Cluj-Napoca, Louis Pasteur Street 6, 400349 Cluj-Napoca, Romania;

[3] Department of Pharmaceutical Analysis, Faculty of Pharmacy, Iuliu Hațieganu University of Medicine and Pharmacy, Louis Pasteur Street 6, 400349 Cluj-Napoca, Romania;

[4] Department of Plant and Animal Cytobiology, Institute of Biology Bucharest, 296 Splaiul Independenței, 060031 Bucharest, Romania;

* Correspondence: iugac@umfcluj.ro

† These authors contributed equally to this work.

Abstract

Leontopodium alpinum Cass. (edelweiss) is recognized as a frequent constituent of anti-aging skin care products, providing increased antioxidant and anti-inflammatory defense. Considering the growing demand and the protected status of edelweiss in many countries, alternative ways of production have been developed, one of them being callus culturing. This study reports the phytochemical composition of a methanolic extract of *L. alpinum* callus cultures, characterized by liquid chromatography coupled to ion-mobility high resolution mass spectrometry (UPLC/IM-HRMS). The methanolic extract exhibited strong free radical scavenging activity (122.19 ± 7.28 [mg AAE/g dw]), while the quantitative evaluation revealed that four major constituents (phenylpropanoid derivatives) represent 57.13% (m/m) of the extract. Consequently, a screening of antiproliferative effects was performed on ten cancer cell lines, representative to prostate, colon, lung and breast cancer, showing inhibition of colony formation in all cases. These results provide a comprehensive phytochemical characterization of *L. alpinum* callus cultures using advanced IM-HRMS, while the *in-vitro* explorations confirmed the potent antioxidant properties of edelweiss which are worth exploring further in cancer prevention.

Keywords: callus cultures, edelweiss, phytochemical characterization, antioxidants, ion-mobility mass spectrometry

Figure S1. Diagnostic product ion - glucaric acid

Figure S2. Diagnostic product ion – quinic acid

Figure S3. Morphological analysis

Figure S4. Colony assay microscopy images and measurements

Figure S1. Diagnostic product ion - glucaric acid

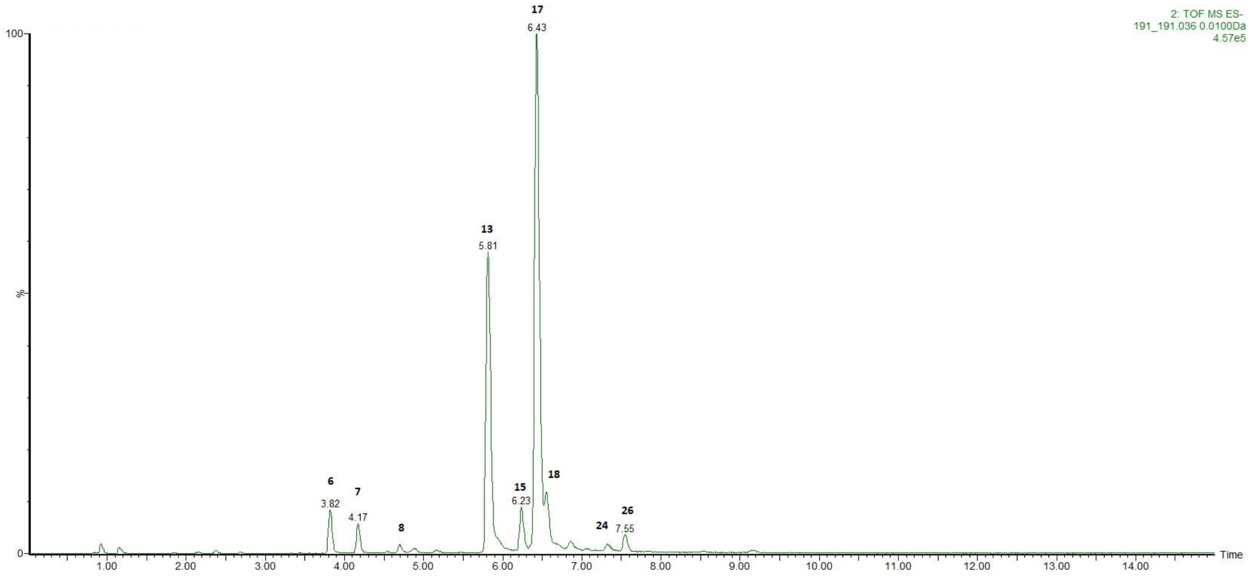

Figure S2. Diagnostic product ion – quinic acid

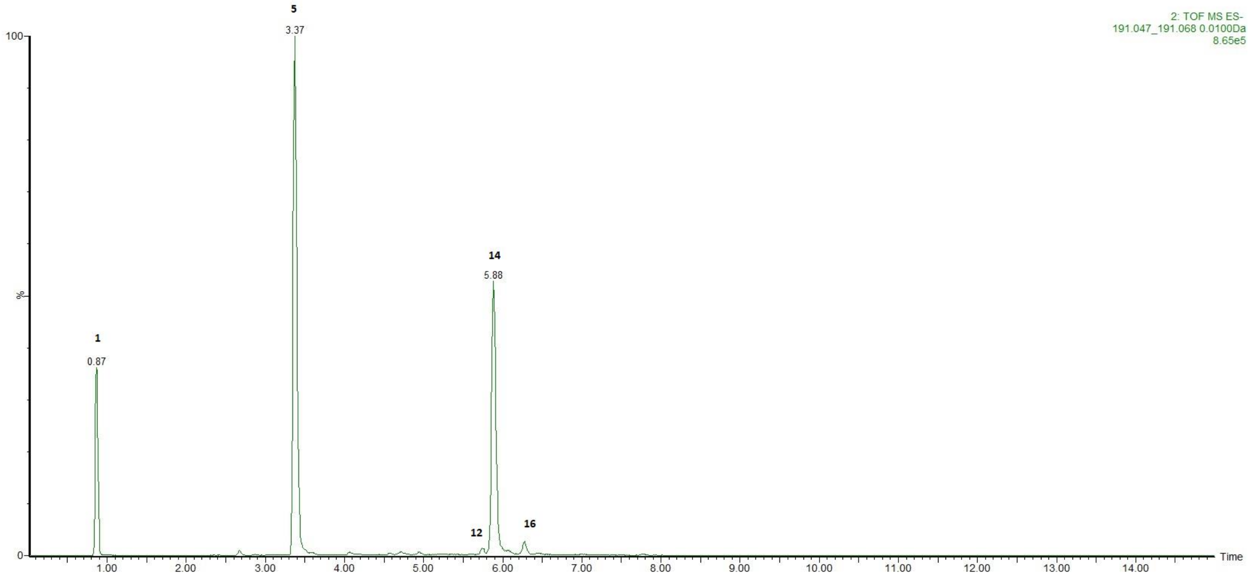

Figure S3. **Morphological analysis.** Effect of *L. alpinum* extract on human normal skin fibroblast, prostate, colon, lung and breast cancer cell lines. Morphological aspects highlighted with DAPI (Blue – for the nucleus) and Phalloidin-FITC (Green – actin filaments). Scale bar of 200 µm, without digital zoom. White arrows – fragmented nucleus; Red arrows – round shaped cells; Turquoise arrows – fragmented cytoskeleton; Green circle – colony size; White circle – multilobular nucleus; Red circle – dividing cells.

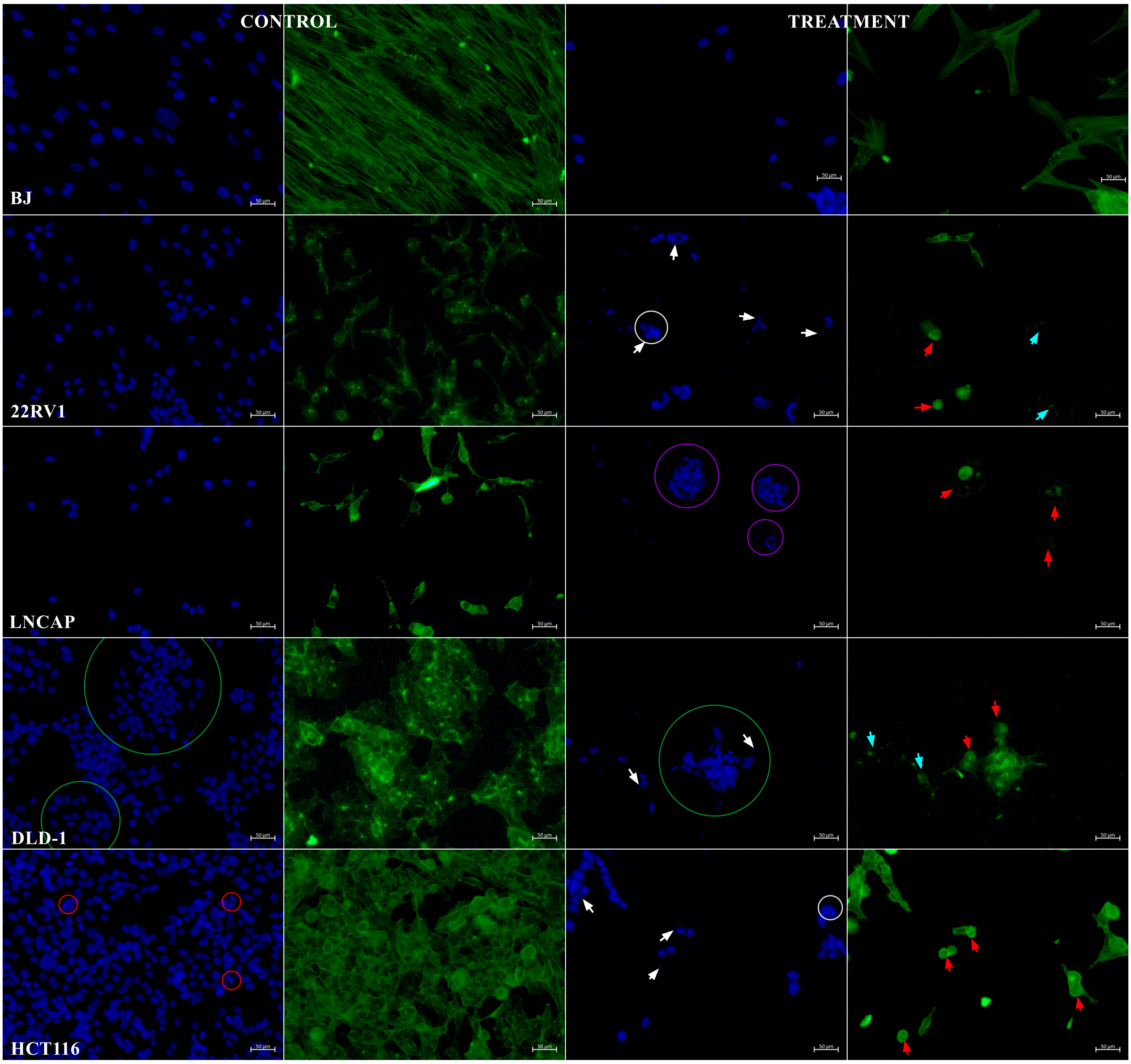

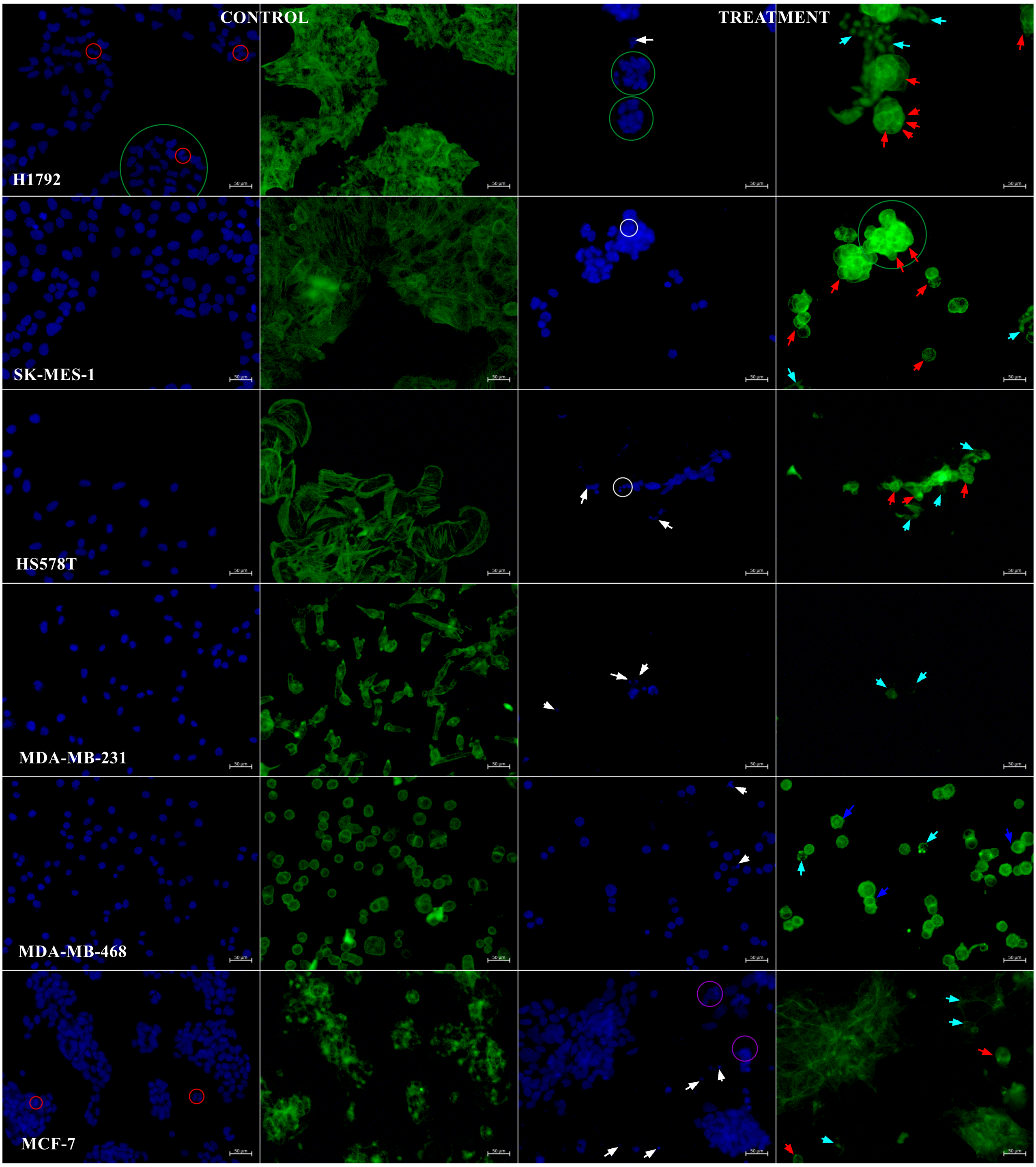
CONTROL
TREATMENT
H1792
SK-MES-1
HS578T
MDA-MB-231
MDA-MB-468
MCF-7

Figure S4. Colony assay. Microscopy images and measurements (n=3)

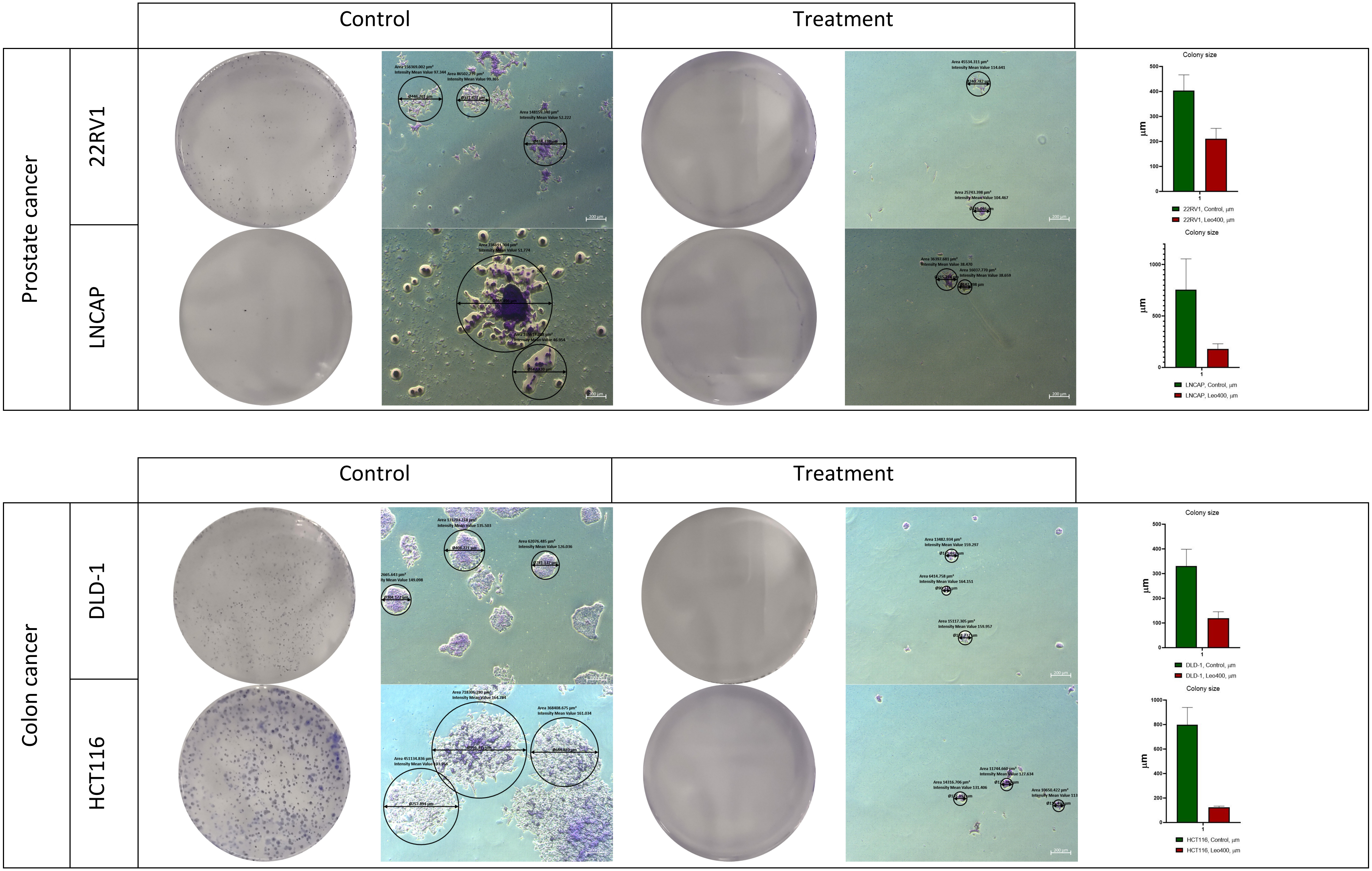

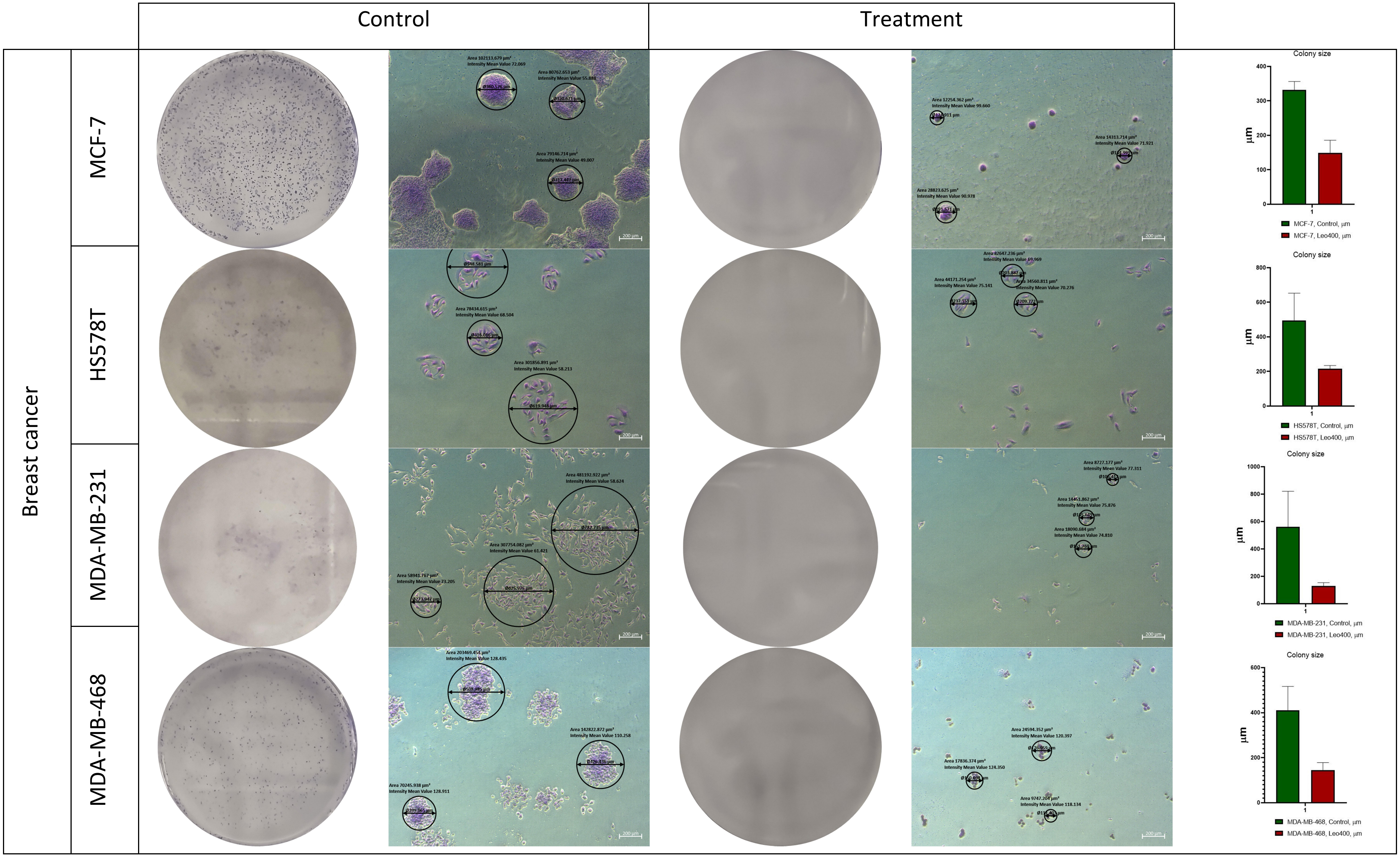

Control
Treatment
Breast cancer
MCF-7
HS578T
MDA-MB-231
MDA-MB-468
Colony size
µm
MCF-7, Control, µm
MCF-7, Leo400, µm
HS578T, Control, µm
HS578T, Leo400, µm
MDA-MB-231, Control, µm
MDA-MB-231, Leo400, µm
MDA-MB-468, Control, µm
MDA-MB-468, Leo400, µm

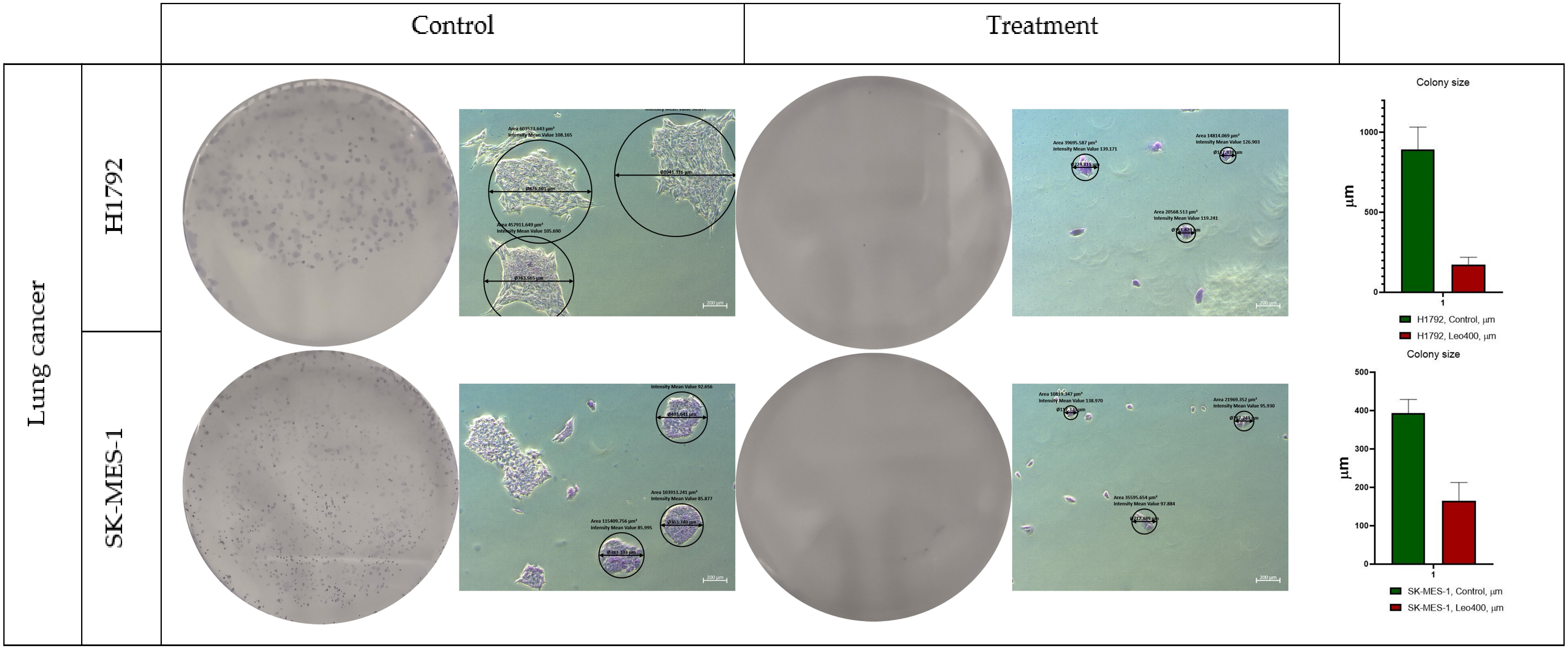
Control
Treatment
Lung cancer
H1792
SK-MES-1
Colony size
µm
1000
500
0
1
H1792, Control, µm
H1792, Leo400, µm
Colony size
500
400
300
200
100
0
µm
1
SK-MES-1, Control, µm
SK-MES-1, Leo400, µm
